# Needle‐free, Liquid Metal‐embedded Electrospray Deposition System for Controlled Microdroplet Printing

**DOI:** 10.1002/advs.202510905

**Published:** 2025-09-26

**Authors:** Chaeyeong Kang, Seokbeom Roh, Hyunjun Park, Sugeun Lee, Taeha Lee, Sang Won Lee, Hansung Kim, Jinsung Park, Gyudo Lee, Insu Park

**Affiliations:** ^1^ Department of MetaBioHealth Sungkyunkwan University Suwon 16419 Republic of Korea; ^2^ Department of Biotechnology and Bioinformatics Korea University Sejong 30019 Republic of Korea; ^3^ Interdisciplinary Graduate Program for Artificial Intelligence Smart Convergence Technology Korea University Sejong 30019 Republic of Korea; ^4^ Department of Biomechatronics Engineering Sungkyunkwan University Suwon 16419 Republic of Korea; ^5^ Department of Biomedical Engineering Yonsei University Wonju 26493 Republic of Korea

**Keywords:** droplet array printing, electrospray deposition, liquid metal integration, microdroplet generation, pdms‐based microfluidics

## Abstract

Electrospray systems have been widely utilized in biomedical, pharmaceutical, and materials science applications due to their ability to enable precise liquid manipulation and efficient ionization. However, conventional electrospray technologies often rely on external ionization components and involve complex system designs, leading to challenges such as clogging, reduced efficiency, and limited reproducibility. This study presents a liquid metal‐embedded electrospray deposition (LM‐ESD) system that incorporates liquid metal within a microfluidic electrospray setup, eliminating the need for additional ionization hardware. By leveraging the intrinsic electric field of the microfluidic chip, the LM‐ESD system simplifies the fabrication process, reduces design complexity, and enhances overall system efficiency. The spray performance is validated using fluorescence microscopy, scanning electron microscopy, atomic force microscopy, Kelvin probe force microscopy, and Raman spectroscopy, all of which confirm the formation of uniform droplet arrays with high reproducibility. This versatile LM‐ESD system shows strong potential for applications in precision printing, thin‐film deposition, and array‐based high‐throughput analysis.

## Graphical abstract

The liquid metal‐embedded electrospray deposition (LM‐ESD) system combines stable droplet spray, clog‐free and recoverable operation, and simplified fabrication in a single polydimethylsiloxane (PDMS) microfluidic platform. By eliminating external electrodes, it offers needle‐free, robust, and scalable droplet array printing, ensuring reproducibility and operational resilience for advanced electrospray application.

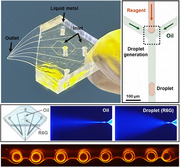

## 1 Introduction

Electrospraying is a versatile technique that employs strong electric fields to disperse liquids into fine aerosol droplets, enabling high‐precision micro‐ and nanoparticle production with minimal sample consumption (typically from nanoliters to microliters); additionally, it has simple operational requirements.^[^ ^1^, ^2^, ^3^, ^4^ ^]^ It has been widely applied in drug delivery systems, microscale droplet generation, and analytical chemistry platforms due to its operational simplicity, high reproducibility, and compatibility with high‐throughput analysis.^[^ ^4^, ^5^, ^6^, ^7^, ^8^ ^]^ Furthermore, electrospray ionization (ESI) mass spectrometry has become a cornerstone in proteomics and pharmaceutical research owing to its exceptional sensitivity and molecular specificity.^[^ ^2^, ^9^, ^10^, ^11^, ^12^ ^]^

Over the past decade, the electrospray field has evolved with the development of advanced modalities aimed at broadening functionality and enhancing system performance. Techniques such as acoustic ESI, desorption ESI, laser ablation ESI, and electrospinning have been employed to improve ionization efficiency, enable surface‐selective deposition, and even facilitate fiber formation. These innovations have extended the applicability of electrospray systems into materials science, biotechnology, and tissue engineering.^[^ ^13^, ^14^, ^15^, ^16^, ^17^, ^18^, ^19^ ^]^ However, most systems still rely on bulky peripheral components (e.g., external electrodes and needle‐based dispensers), posing challenges to device miniaturization, continuous operation, and system integration. Notably, needle clogging and unstable spray modes remain persistent issues, particularly in microfluidic environments and when handling viscous or complex fluids.

To overcome these limitations, significant efforts have been made to integrate electrospray functionality directly within microfluidic platforms. Notable approaches include embedding platinum‐sputtered electrodes along channel walls and fabricating electrospray‐compatible nozzles using SU‐8 photolithography in conjunction with capillary electrophoresis‐ESI systems. ^[^ ^8^, ^16^, ^17^, ^18^, ^20^, ^21^ ^]^ While these strategies offer partial integration, they still involve multi‐step fabrication, high‐voltage wiring external to the chip, and fragile component alignment—all of which hinder compactness and robustness, especially for portable or disposable applications. Furthermore, the reliance on cleanroom‐based microfabrication and rigid materials such as glass or silicon limits design flexibility, scalability, and widespread adoption.^[^ ^22^, ^23^, ^24^ ^]^ These challenges underscore the demand for simplified, robust, and fully integrated electrospray systems that can be embedded within microfluidic devices without compromising performance or manufacturability. In previous studies, integrated on‐chip electrodes for electrospray generation have been demonstrated in both polydimethylsiloxane (PDMS)‐ and glass‐based microfluidic systems, for example, a monolithically integrated platinum emitter in a glass chip for HPLC–droplet–MS coupling^[^ ^25^ ^]^ and microfluidic‐MS platforms for high‐throughput bioassays.^[^ ^26^ ^]^ While these works confirmed the feasibility of on‐chip ESI, they relied on rigid substrates and complex micromachining, which limited robustness, scalability, and ease of fabrication. These challenges highlight the inherent difficulty of achieving truly compact, stable, and manufacturable electrospray systems.

In this study, we present a novel liquid metal‐embedded electrospray deposition (LM‐ESD) system that directly integrates liquid metal electrodes into PDMS‐based microfluidic platforms. Unlike conventional systems that rely on external electrodes or needle‐based emitters, the LM‐ESD system features a fundamentally simplified and fully integrated architecture. ^[^ ^27^ ^]^ By leveraging the intrinsic conductivity of the embedded liquid metal, the system generates a stable and uniform electric field within the microchannel, eliminating the need for external components.^[^ ^28^, ^29^ ^]^ This built‐in integration streamlines fabrication by removing post‐fabrication electrode assembly, reduces overall system complexity, and enhances spray stability and reproducibility. Stable voltage delivery to the emitter is essential for consistent electrospray performance, and the conformal integration of eutectic gallium–indium (EGaIn) electrodes ensures both voltage stability and uniform field distribution. Unlike previous embedded electrode designs developed mainly for mass spectrometry interfaces,^[^ ^30^, ^31^ ^]^ our LM‐ESD system approach enables precise, on‐demand droplet printing on substrates with tunable droplet size, spacing, and periodicity, which were not demonstrated in prior MS‐only systems. Furthermore, the LM‐ESD system supports scalability and high‐throughput processing by avoiding fragile component insertion or additional bonding steps, thereby improving mechanical robustness and enabling compatibility with wafer‐scale batch fabrication.

The performance of the LM‐ESD system was validated through comprehensive droplet array characterization using fluorescence microscopy, scanning electron microscopy (SEM), atomic force microscopy (AFM), Kelvin probe force microscopy (KPFM), and Raman spectroscopy. The system consistently demonstrated stable droplet array formation and high spatial uniformity across various flow conditions. Notably, it enabled precise control over droplet placement and size, which is critical for accurate array printing.^[^ ^32^ ^]^ These results highlight the strong potential of the LM‐ESD system for applications requiring high pattern fidelity, such as semiconductor thin‐film deposition and single‐cell localization in biomedical assays. In summary, the LM‐ESD system represents a robust and versatile solution for droplet‐mediated deposition and printing, offering substantial promise for both fundamental research and high‐precision industrial applications.

## 2 Result and Discussion

### 2.1 Fabrication and Construction of LM‐ESD System

**Figure 1:**
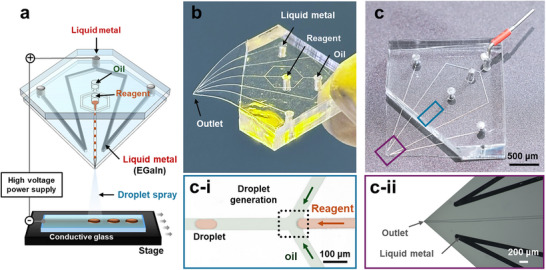
Droplet‐based microfluidic system for electrospray generation using liquid metal. a) 3D schematic of the device configuration, showing independent inlets for reagent (orange) and oil (green), and a fixed reservoir for liquid metal (EGaIn). Droplets are formed via pressure‐driven flow focusing and sprayed onto a conductive glass substrate under high voltage. b) Photograph of the assembled microfluidic chip with separate channels guiding the oil, reagent, and liquid metal. c) Photograph of the microfluidic device. The blue box highlights the droplet generation region, while the purple box indicates the outlet region containing the outlet and liquid metal. (c‐i) Enlarged microscopic image of the droplet generation junction (blue box), showing discrete microscale droplets formed by the oil and reagent streams. c‐ii) Enlarged microscopic image of the outlet region (purple box), showing the outlet and confined liquid metal.

The LM‐ESD system was designed with separate channels for the injection of internal liquid metal, oil, and reagents, thereby eliminating the need for external electrodes. The symmetrical placement of the liquid metal allows for stable droplet ejection. In addition, a high voltage was applied between the liquid metal and the conductive glass substrate, and an Arduino‐controlled motor was implemented beneath the substrate to enable constant‐speed horizontal motion (**Figure** **1**a). This straightforward design allows direct electrospray through the system's outlet without complex auxiliary configurations. The device employed EGaIn as a built‐in electrode, allowing electrospraying to occur directly through the outlet by simply applying a high voltage and translating a conductive glass substrate beneath the outlet. The device was fabricated entirely from PDMS using a conventional lithography process (Figure 1b,c) and incorporated a flow‐focusing geometry for controlled droplet generation. In particular, the PDMS layer at the outlet was made thinner than 200 µm to ensure stable droplet jetting. As illustrated in Figure S1 (Supporting Information), a uniform cone‐jet mode was observed with an outlet thickness of 200 µm, whereas a thicker outlet (5 mm) led to unstable multijet behavior. This phenomenon was attributed to outlet thickness rather than the distance to the conductive substrate (Movie S1, Supporting Information). Thinner nozzle tips enhance electric field concentration, which stabilizes electrospraying and facilitates the formation of finer droplets with a higher surface‐to‐volume ratio.^[^ ^33^, ^34^ ^]^

Figure 1c–i further shows that the reagent and oil phases were introduced through separate inlets, where microscale water‐in‐oil droplets were generated via hydrodynamic shearing at the junction by the continuous oil phase (Figure S2, Supporting Information). The boiling points of the oil and the Rhodamine 6G (R6G solution used in the spraying process are ≈128 °C and 70 to 80 °C, respectively. When only the R6G solution with a relatively low boiling point was continuously sprayed onto the substrate, the deposited droplets did not evaporate but gradually coalesced into larger water droplet‐like structures, as illustrated in Figure S3 (Supporting Information). Taken together with these experimental observations and previously reported studies on electrospray coating under various parameters, the present results demonstrate that the proposed LM‐ESD system operates in a wet‐spray mode on the substrate, thereby facilitating droplet formation.^[^ ^35^, ^36^, ^37^ ^]^

By adjusting the flow rates of the reagent and oil, both droplet size and generation frequency could be precisely controlled, resulting in monodisperse droplet formation with consistent size and shape. The key innovation lies in the use of liquid metal electrodes, which were injected into side channels flanking the outlet and remained stably confined (Figure 1c(ii)). This configuration provides a conformal, non‐invasive electrode interface that avoids problems associated with needle insertion or surface electrode attachment, such as channel damage and inconsistent contact.

To validate the advantages of the LM‐ESD system, we compared it with conventional electrospray setups that employ either inserted metal needles or printed circuit board (PCB)‐based electrodes (Table S1, Supporting Information). While needle‐based systems offer focused spraying from narrow outlets, they risk damaging the PDMS and lack long‐term operational stability. Meanwhile, PCB‐printed electrodes are less invasive, but their thin glass substrates are fragile, and their reliance on conductive tape introduces variability in the applied voltage. Although increasing substrate thickness can reduce mechanical fragility, it also increases resistance, thereby reducing electrical efficiency.

In contrast, the LM‐ESD system successfully implements a needle‐free, PDMS‐based design incorporating liquid metal electrodes, thereby eliminating the need for external electrode insertion or attachment. This configuration enables a simple PDMS layer assembly process for fabrication and improves system reliability, without requiring lithography, electrode printing, or metal patterning.

To evaluate the physical robustness of the LM‐ESD device, two sets of stability tests were performed: electrical resistance measurements under mechanical deformation, including stretching and bending (Figure S4, Supporting Information), and repeated reinjection of liquid metal (Figure S5, Supporting Information). After assembling the LM‐ESD system and injecting EGaIn, the electrode resistance was measured under various mechanical conditions (Figure S4a, Supporting Information). In the undeformed state, the resistance obtained by the two‐point probe method averaged 6.62 ± 0.23 Ω (Figure S4b, Supporting Information). When the device was stretched horizontally and vertically, the resistance values were 7.08 ± 0.75 and 6.55 ± 0.25 Ω, respectively (Figure S4c,d, Supporting Information). Similarly, upward and downward bending yielded resistances of 6.48 ± 0.24 and 6.40 ± 0.24 Ω (Figure S4e,f, Supporting Information). All values under various mechanical deformation conditions showed no statistically significant differences (Figure S4g, Supporting Information). These results confirm that the LM‐ESD system maintains stable electrical properties under diverse mechanical stresses, experimentally validating its structural integrity.

Furthermore, the stability of reinjection was assessed by sequentially replacing the liquid metal and recording the resistance over three cycles (Figure S5a, Supporting Information). The measurements yielded a highly consistent resistance with an RSD of 1.55% (Figure S5b, Supporting Information), indicating negligible variation even after repeated replacement of the electrode material. These findings highlight the superior practicality and operational stability of liquid metal–based electrodes compared to conventional solid metal counterparts. In addition, as demonstrated in Movie S2 (Supporting Information), the LM‐ESD system allows simple manual pressing of the PDMS channel to remove residual blockages, thereby ensuring recoverable spraying operation and underscoring the platform's robustness against clogging. To further clarify these advantages, we compared the developed LM‐ESD system with previously reported ESI cone‐jet spraying platforms (Table S2, Supporting Information). This comparison revealed that the LM‐ESD uniquely integrates room‐temperature operation, mechanical resilience, voltage stability, and patterning capability features not collectively validated in earlier studies.

### 2.2 Analysis of Droplet Size and Frequency Based on Flow Rate

**Figure 2:**
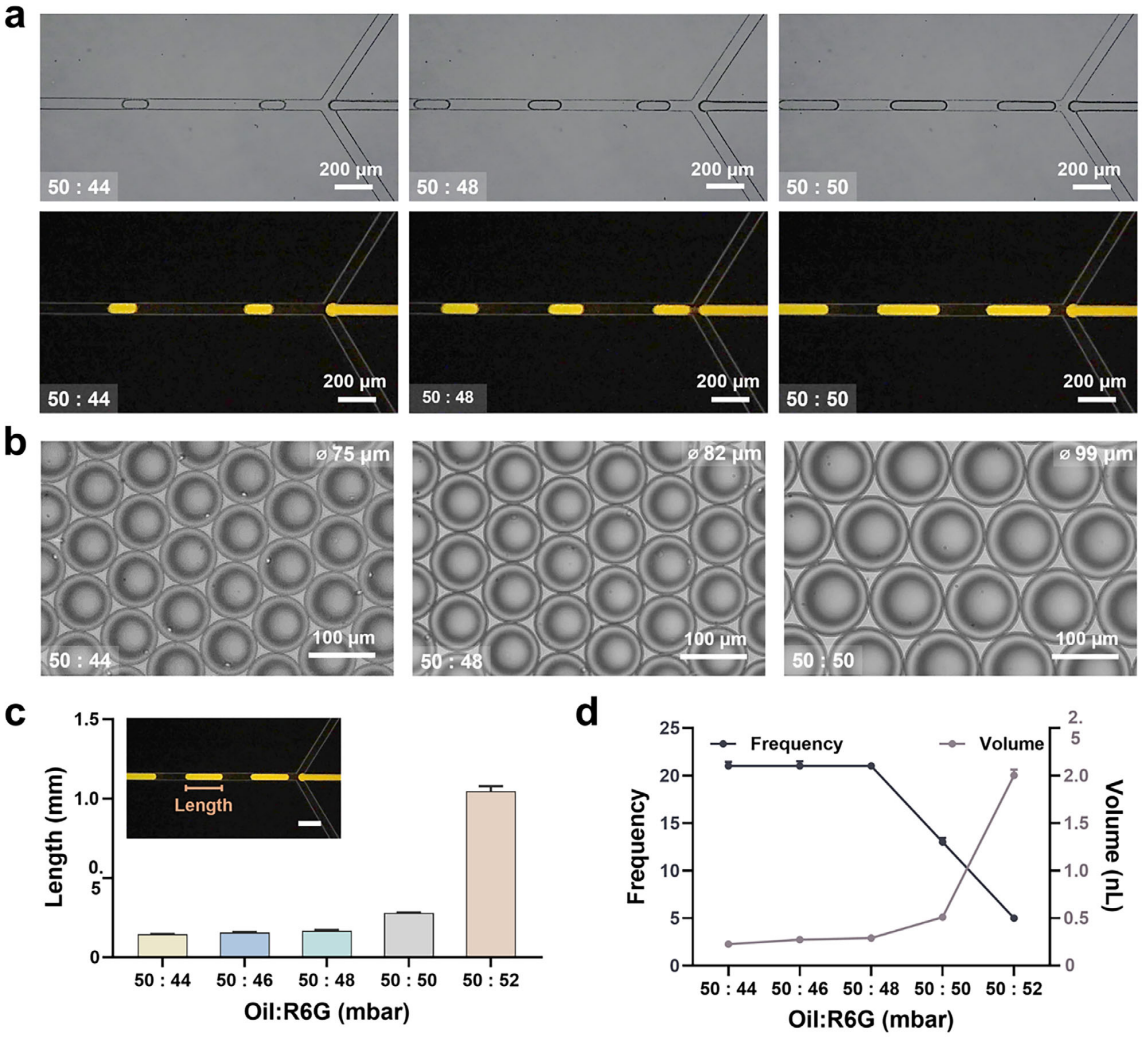
Characterization of droplet size and generation frequency at varying oil‐to‐reagent pressure ratios. a) Bright‐field (top) and fluorescence (bottom) images of droplets formed at oil‐to‐R6G solution pressure ratios of 50:44, 50:48, and 50:50 (mbar). Increasing reagent pressure produced visibly elongated droplets. Scale bars: 200 µm. b) Optical images showing droplet diameters under corresponding flow conditions. Mean diameters were 75, 82, and 99 µm, respectively. Scale bars: 100 µm. c) Droplet length as a function of the oil‐to‐R6G solution pressure ratio. Inset: fluorescent image illustrating droplet length within the microfluidic channel. Scale bar: 200 µm. d) Quantitative analysis of droplet generation frequency (left axis) and estimated droplet volume (right axis) under different flow conditions. Higher reagent pressure led to increased droplet volume and decreased generation frequency.

To characterize droplet behavior as a function of flow rate using the LM‐ESD system, a series of controlled experiments was conducted by varying the pressure ratio between the continuous oil phase and the R6G dispersed phase. Fluorescence imaging was employed to enhance visualization accuracy (**Figure** **2**a). Droplet formation in the LM‐ESD system was driven by hydrodynamic shear at the flow‐focusing junction, with oil serving as the continuous phase. The oil pressure was fixed at 50 mbar to ensure stable baseline conditions, while the reagent (R6G) pressure was varied from 44 to 52 mbar in 2 mbar increments.^[^ ^38^ ^]^ Reagent pressures below 50 mbar showed excessive sensitivity to pneumatic fluctuations, leading to unstable droplet formation, whereas higher oil pressures (>50 mbar) caused overly rapid droplet generation, complicating image‐based analysis.^[^ ^39^, ^40^ ^]^

For each pressure condition, droplet generation frequency was quantified by counting the number of droplets passing through a defined junction per second. Droplet volume was estimated from captured images using a geometric approximation: a cylindrical core with hemispherical end caps. Given the channel dimensions (50 µm width and depth), this simplified model provided a reliable estimation of actual droplet volume under laminar flow. Bright‐field and fluorescence images were acquired using standard and Tetramethylrhodamine (TRITC) filters, cropped into frame sequences, and analyzed (Figure 2a). A clear increase in droplet size was observed with increasing reagent pressure (Movie S3, Supporting Information). Fluorescence images revealed that droplet diameter increased from 75 µm at a 50:44 (oil:R6G) pressure ratio to 99 µm at 50:50 (Figure 2b). Droplet elongation at the outlet was further assessed by measuring droplet lengths, yielding average values of 144 ± 2.23, 156 ± 3.09, 167 ± 5.02, 278 ± 3.19, and 1047 ± 31.42 µm for reagent pressures of 44, 46, 48, 50, and 52 mbar, respectively (Figure 2c).

Figure 2d illustrates the relationship between pressure ratio, droplet frequency, and estimated volume. As reagent pressure increased relative to oil pressure, the effective shear force at the junction decreased, resulting in slower encapsulation and larger droplet formation. This inverse relationship between droplet volume and frequency is consistent with classical shear‐dominated droplet generation in microfluidic systems. At lower reagent flow rates, higher shear promoted faster pinch‐off and increased droplet frequency, resulting in smaller droplets. This tunability demonstrates the LM‐ESD system's capacity for precise droplet size control under simple pressure‐driven operation.

### 2.3 Analysis of Droplet Generation Rate and Droplet Spray Sequence

**Figure 3:**
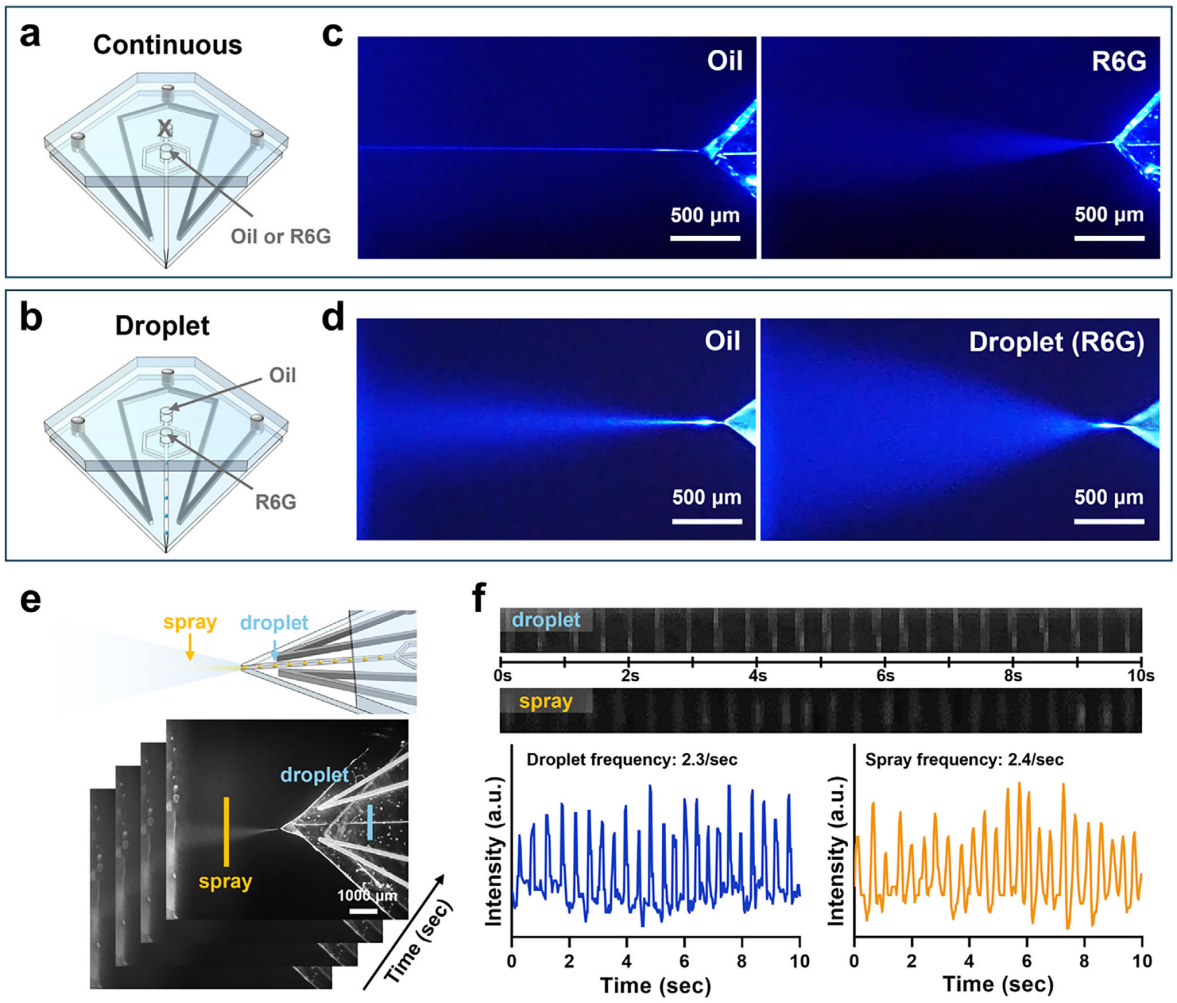
Comparison of continuous and droplet‐based electrospray modes using the LM‐ESD system. a,b) Schematic illustrations of the electrospray setup using a single inlet for continuous phase injection (a) and dual inlets for droplet generation (b), with oil and R6G serving as the carrier and dispersed phases, respectively. c,d) Optical images of electrospray plumes in continuous (c) and droplet‐based (d) configurations, illustrating distinct spray patterns. Scale bars: 500 µm. e) Sequential images showing the dynamic transition between spray and droplet regimes. Yellow and cyan bars indicate regions of interest used to extract grayscale intensity for frequency analysis of droplet spray and droplet generation within the channel, respectively. Scale bar: 1000 µm. f) Temporal grayscale intensity profiles derived from time‐lapse image sequences. The calculated generation frequencies are 2.3 s^−1^ for the droplet mode and 2.4 s^−1^ for the continuous spray mode.

To investigate the morphological characteristics of droplet spray patterns in the LM‐ESD system, we examined the electrospray behavior of oil and R6G solution under both continuous and droplet generation modes. Additionally, the temporal correlation between droplet formation and spray emission was analyzed. In the single‐inlet configuration, either oil or R6G solution was individually introduced into the device to isolate and characterize their respective spray profiles (**Figure** **3**a). As shown in Figure 3c, oil generated a narrow, collimated spray, whereas R6G produced a broader, more radially dispersed plume. These results served as visual baselines to distinguish oil and droplet phases during dual‐inlet operation.

Under dual‐inlet conditions, both oil and R6G solution were introduced simultaneously, producing periodic droplets via flow focusing (Figure 3b). The resulting spray exhibited alternating morphological features corresponding to those observed in the single‐inlet experiments (Figure 3d): straight segments were associated with oil ejection, while radial plumes indicated the presence of droplet‐laden sprays. This confirmed that the spray morphology reliably reflected the internal fluid sequencing during operation.

To further characterize the synchronization between droplet formation and spray emission, time‐lapse images were recorded in both the droplet generation region and the downstream spray zone (Figure 3e). Grayscale intensity analysis was performed on two defined regions of interest—one aligned with the droplet channel (cyan) and the other aligned with the spray plume (yellow). Intensity fluctuations over time were extracted and plotted to visualize temporal patterns (Figure 3f). The results showed synchronized intensity peaks in both regions, with each peak corresponding to a droplet generation event and its subsequent spray emission. Droplet generation occurred at an average frequency of 2.3 events per second, while spray events occurred at 2.4 events per second. The near‐identical frequencies and uniform peak spacing indicated that droplets were ejected immediately after formation without accumulation or temporal delay. These findings demonstrate that the LM‐ESD system enables stable and temporally synchronized droplet array spraying. Notably, the droplet generation frequency was virtually unchanged with or without voltage application (14.8 vs 14.6 s^−1^, Figure S6, Supporting Information), indicating that the embedded LM electrodes do not perturb intrinsic droplet dynamics and thereby support the operational robustness of the LM‐ESD system.

### 2.4 Optimization of Droplet Spraying Conditions

Following the validation of uniform droplet generation and synchronized ejection, the droplet spray characteristics of the LM‐ESD system were further evaluated under key operational parameters, including the outlet‐to‐substrate distance, outlet diameter, and magnitude of the applied voltage. Three metrics were used: spray angle, Taylor cone size, and droplet atomization size. Stronger electric fields result in more collimated and parallel sprays, whereas weaker electric fields result in broader dispersion. Therefore, the spray angle is an indirect indicator of the spraying behavior under different electric fields. The droplet spray size refers to the spatial dispersion of both the ejected droplet and its surrounding medium (oil sheath), which is strongly influenced by the electric field strength between the outlet and the substrate.^[^ ^41^ ^]^ A higher electric field leads to more focused and collimated spraying, while a weaker field results in broader dispersion. Thus, the spray size serves as an indirect indicator of field‐dependent ejection behavior. In contrast, the Taylor cone size reflects the interfacial tension balance at the nozzle tip: under weaker fields, surface tension dominates, leading to larger cones; under stronger fields, the cone contracts, enabling more efficient atomization.^[^ ^42^ ^]^

**Figure 4:**
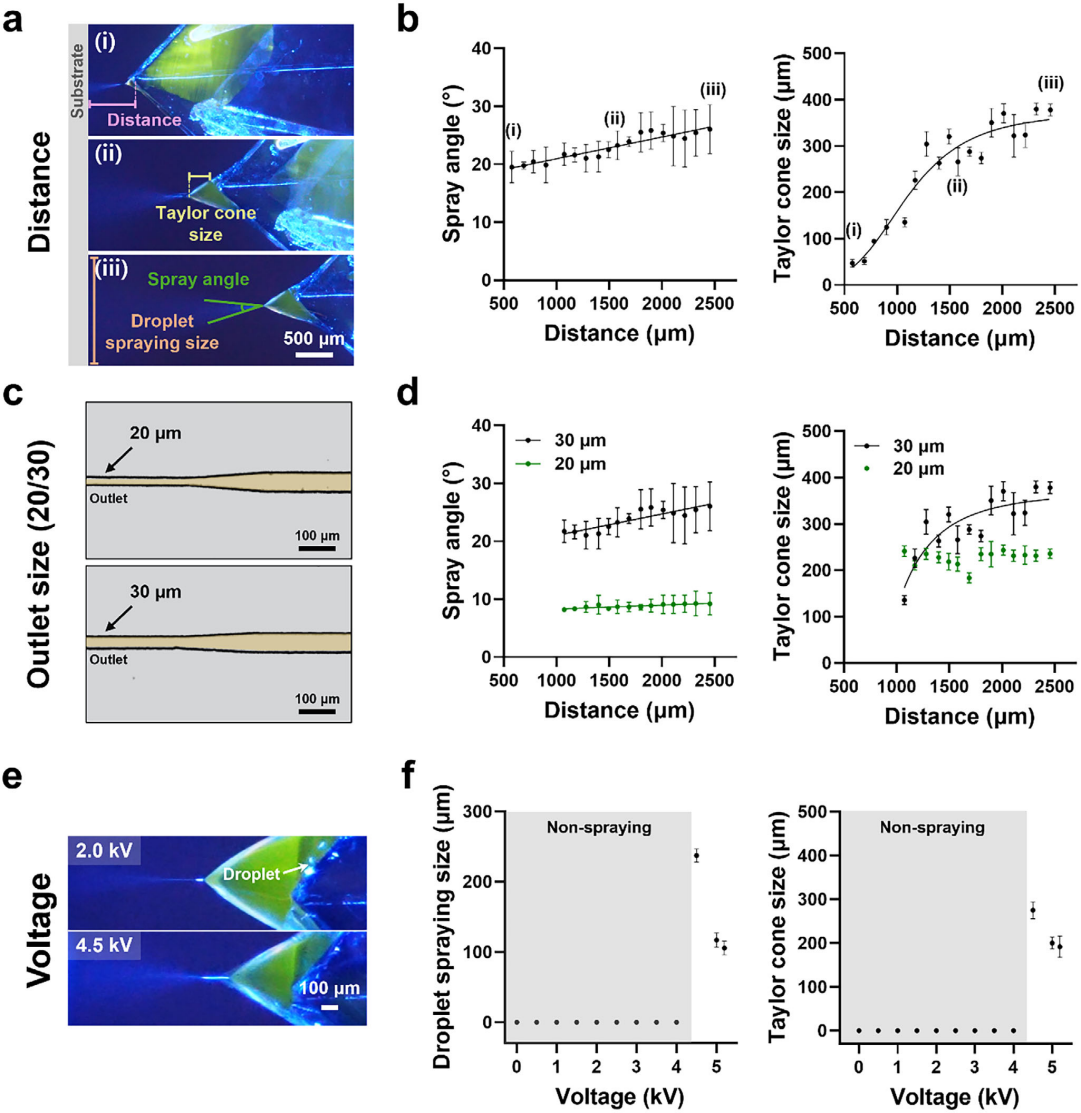
Analysis of droplet spray size and Taylor cone size under various conditions. a) Schematic illustration indicating the measured parameters: spray distance (purple), Taylor cone size (yellow), spray angle (green), and droplet spray size (orange). b) Graph showing the correlation between spray distance and both spray angle (R^2^ = 0.8888) and Taylor cone size (R^2^ = 0.8966). c) Optical images of nozzles with different outlet sizes (20 and 30 µm). d) Graphical analysis of spray angle and Taylor cone size as a function of outlet size. The spray angle exhibited R^2^ = 0.7835 (30 µm) and R^2^ = 0.7300 (20 µm). The Taylor cone size exhibited R^2^ = 0.6766 (30 µm), whereas under the 20 µm outlet condition, it remained nearly constant with distance (mean = 226.6 µm, 95% CI: 223.1–230.1, n = 140) and was therefore summarized without curve fitting. The spray angle was analyzed by linear regression, while the Taylor cone size was analyzed using a sigmoidal (four‐parameter logistic) model. e) Optical images showing the effect of applied voltage (2.0 and 4.5 kV) on droplet formation. f) Relationship between applied voltage and both droplet spray size (left) and Taylor cone size (right), illustrating a distinct transition from non‐spraying to electrospray mode above 4.0 kV.

**Figure** **4**a illustrates the dependence of droplet spray and Taylor cone geometry on outlet‐to‐substrate distance. To assess this parameter, the gap was varied from ≈580 to 2450 µm under constant flow rate and voltage conditions. As shown in Figure 4b, the variation in the spray angle with respect to the distance between the LM‐ESD system and the substrate exhibits a gradual but linear increase. This result can be attributed to the fundamental operating principle of the LM‐ESD system, which relies on the generation of droplet spraying through electric fields. When the substrate is positioned closer to the outlet, the stronger electric field accelerates the charged droplets along a relatively straight trajectory, resulting in a narrower spray angle. On the other hand, as the distance increases, the electric field strength decreases, leading to a broader electric field distribution around the spray outlet and consequently a wider dispersion of droplets, which increases the spray angle.^[^ ^43^ ^]^ In addition, the variation in Taylor cone size with respect to the system–substrate distance was analyzed with a sigmoidal curve‐fitting model, which revealed a nonlinear saturation behavior. This indicates that the cone size does not continuously increase with distance but rather reaches a saturation point, demonstrating a nonlinear dependency. This phenomenon can be attributed to the weakening of the electric field at greater distances, where surface tension becomes dominant, resulting in the formation of more rounded and enlarged cone shapes (Movie S4, Supporting Information).

Figure 4c presents the effect of outlet diameter (20 µm vs 30 µm) on spray behavior. All other experimental variables were fixed, and only the distance between the LM‐ESD systems, each with different nozzle diameters—and the conductive substrate was varied. Subsequently, the spray angle and Taylor cone size were analyzed for each nozzle diameter condition. As shown in Figure 4d, the spray angle increased with increasing distance for both nozzles; however, the 20 µm nozzle consistently maintained a relatively smaller spray angle. Likewise, the Taylor cone size exhibited minimal variation and a stable trend under the 20 µm outlet condition. These observations suggest that a smaller nozzle diameter enhances the localization of the electric field, thereby improving the overall stability of the system.

Finally, an optimization study was conducted to examine the influence of applied voltage on electrospray behavior (Figure 4e). No droplet or oil spraying was observed between 0 and 4 kV. At 1.5 kV, a Taylor cone appeared but without actual emission. Oil spray was observed at 2 and 4 kV, but the aqueous droplets failed to exit the nozzle and instead retracted along the channel walls. Stable and reproducible droplet emission was only observed at 4.5 kV and above (Movie S5, Supporting Information). Figure 4f summarizes this voltage‐dependent behavior: voltages below 4 kV represent a non‐spraying regime, characterized by partial or suppressed emission. Above 4 kV, both the droplet spray size and Taylor cone size decreased with increasing voltage, confirming that these metrics are directly influenced by the magnitude of the applied electric field. Additionally, for spray systems used in micro‐printing and deposition, an operating time of only a few seconds to less than one minute is generally sufficient for practical applications.^[^ ^44^ ^]^ Nevertheless, the fabricated LM‐ESD system was evaluated under extended operation; continuous spraying of the R6G solution was stably maintained for over three minutes until the prepared solution was completely depleted (Movie S6, Supporting Information).

### 2.5 Analysis of Droplet Array Spray

**Figure 5:**
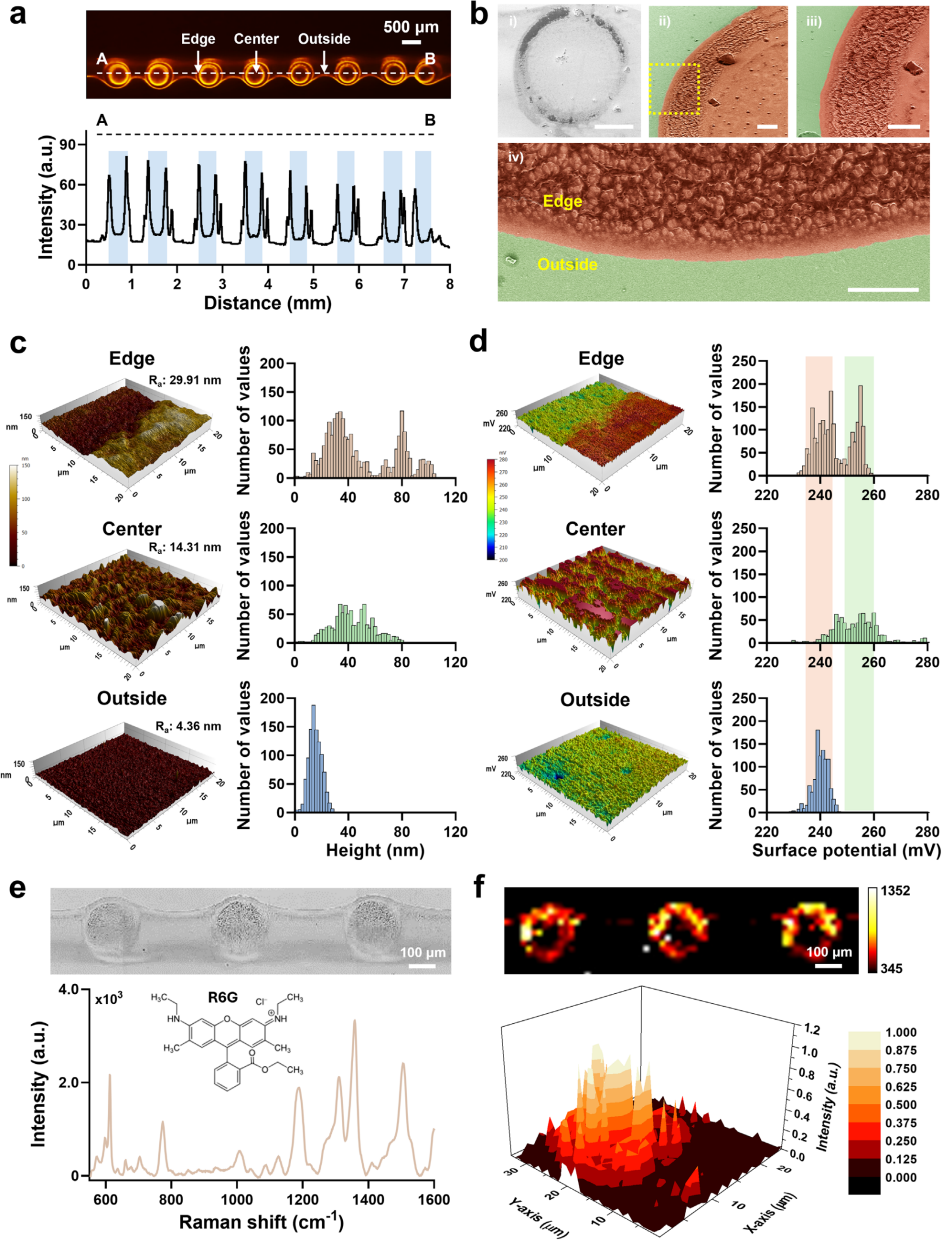
Characterization of droplet array deposition generated by the LM‐ESD system. a) Fluorescence image of the deposited droplet array, highlighting spatial zones including the edge, center, and outside regions. The white dashed line indicates the cross‐sectional profile of fluorescence intensity, with individual droplets marked in yellow. b) SEM images of a single deposited droplet: (i) overall droplet morphology (scale bar: 100 µm), (ii) magnified view of the droplet edge (scale bar: 10 µm), (iii) intermediate magnification (scale bar: 10 µm), and (iv) high‐resolution image showing the edge‐to‐outside transition (scale bar: 10 µm). c) AFM‐based topography analysis of edge, center, and outside regions, with corresponding surface roughness values (R_a_). d) KPFM surface potential maps, with histograms indicating distribution differences among the edge, center, and outside regions. e) Raman microscopy image of the droplet array (top) and Raman spectrum of R6G acquired from the deposited region (bottom). f) Raman mapping of a single droplet, shown as both a 2D projection and a 3D intensity plot, confirming the spatial localization of the R6G signal.

Building upon the prior evaluation of the effects of distance, outlet size, and voltage on droplet spray formation, we further investigated the spatial uniformity and deposition stability of the droplet arrays generated by the LM‐ESD system. As shown in **Figure** **5**a, fluorescence microscopy confirmed the formation of a uniform droplet array with clearly defined spatial regions. The cross‐sectional fluorescence intensity profile demonstrated consistent droplet morphology and periodic spacing across the substrate, indicating stable and repeatable operation. Complementary measurements (Figure S7, Supporting Information) confirmed an average droplet diameter of ≈350 µm, further validating the system's ability to produce size‐controlled droplets. Notably, the droplet size could be tuned by adjusting electrospray parameters, offering versatility for different applications.

Fluorescence distribution analysis revealed that R6G predominantly accumulated at the droplet periphery, forming a ring‐like emission pattern (Figure 5a). This behavior is attributed to the coffee‐ring effect, wherein solutes migrate outward during solvent evaporation due to capillary flow. Despite partial ionization induced by high‐voltage spraying, the solute redistribution observed suggests that electrohydrodynamic forces did not suppress evaporation‐driven transport. Similar radial migration behavior has been reported in electrospray systems, confirming that capillary‐driven flow remains the dominant mechanism for solute positioning within dried droplets under the LM‐ESD system operating regime.^[^ ^45^ ^]^

To examine the morphological features of the deposited droplets, SEM was used (Figure 5b). The low‐magnification image (Figure 5b(i)) showed the overall droplet shape, while higher‐magnification images (Figure 5b(ii,iii)) revealed differences in surface texture across droplet regions. The enlarged edge view (Figure 5b(iv)) showed a gradual transition in surface features from the interior toward the edge, indicating possible differential material accumulation and evaporation dynamics that influenced the final deposition profile.

AFM was employed to further analyze the topological and electrostatic properties of the dried droplets, yielding both topography (Figure 5c) and surface potential (Figure 5d) maps. The topography images revealed distinct height variations across the droplet regions, with statistical analysis showing two prominent peaks at the edge. The measured surface roughness values were 29.91 nm at the edge, 14.31 nm at the center, and 4.36 nm in the outer region, indicating increased roughness due to R6G deposition. The KPFM surface potential maps showed two distinct potential peaks at the droplet edge, reflecting localized charge accumulation. Interestingly, the potential values at the center and outside regions were close to those of the edge peaks, suggesting a unique and spatially modulated charge distribution around the droplet. While fluorescence and height profiles confirmed solute accumulation at the edge, the KPFM images did not show a proportional increase in surface potential at those locations. As validated on both indium tin oxide (ITO) and gold substrates (Figure S8, Supporting Information), elevated surface potential values were observed at the edge and center, but were substantially lower in the outer region. This decoupling between material accumulation and electrostatic localization suggests that surface charge distribution is influenced by additional processes. These may include charge neutralization upon contact with conductive substrates,^[^ ^46^, ^47^ ^]^ lateral ionic diffusion during drying,^[^ ^48^, ^49^ ^]^ or the formation of neutral R6G aggregates at the droplet periphery. ^[^ ^50^, ^51^ ^]^ Together, these findings indicate that charge and mass transport during electrospray deposition are governed by distinct and complex physical mechanisms.

Several factors may account for this decoupling. First, upon contact with conductive substrates, partial or complete charge dissipation may occur through electron‐ion recombination, facilitating rapid charge neutralization.^[^ ^52^ ^]^ Second, ionized species may undergo lateral redistribution during the drying process, resulting in a more uniform electrostatic profile across the droplet footprint.^[^ ^53^ ^]^ Third, the formation of charge‐neutral R6G aggregates at the droplet edge is supported by spectroscopic evidence.^[^ ^54^ ^]^ Specifically, concentration‐dependent dimerization of R6G characterized by hypsochromic shifts in absorption spectra, confirms the preferential formation of neutral dimers at high local concentrations.^[^ ^55^ ^]^ These aggregates, due to excitonic coupling, exhibit minimal net charge, which is consistent with the observed low surface potential values at the edges despite visible material accumulation. Collectively, these results highlight a decoupling of mass and charge transport during electrospray deposition, suggesting that electrostatic interactions, molecular aggregation, and solvent evaporation jointly influence surface potential distributions.

To further verify the compositional integrity of the deposited droplets, Raman spectroscopy analysis was performed. A uniform optical image of a deposited R6G droplet is shown in the upper panel of Figure 5e, revealing visible differences between the edge, center, and outer regions. To quantify this observation, Raman spectra were collected across the droplet. The characteristic peaks of R6G were observed at 614, 779, 1356, and 1506 cm^−1^ with the 1506 cm^−1^ peak used as a reference for mapping. Based on spectra extracted using WiRE 5.1 software, mapping images were generated at all four characteristic peaks using GraphPad Prism 9 (Figure S9a, Supporting Information). As shown in Figure 5f, the Raman intensity was highest at the droplet edge, consistent with the coffee‐ring effect. Additional morphological and Raman mapping data across 17 droplets deposited by the LM‐ESD system are provided in Figure S9b (Supporting Information), supporting the reproducibility and uniformity of the deposition. A 3D Raman intensity map of a selected droplet region (bottom panel, Figure 5f) further confirmed that the maximum Raman signal occurred at the edge, reinforcing the conclusion that solute accumulation is driven by capillary flow during solvent evaporation.^[^ ^56^, ^57^ ^]^

## 3 Conclusion 

We designed, developed, and demonstrated a liquid metal‐based electrospray deposition (LM‐ESD) system that integrates liquid metal electrodes within a microfluidic chip, enabling a compact and efficient array‐spraying platform. The LM‐ESD system exhibited precise and uniform droplet generation at the nanoliter scale, ensuring high reproducibility and operational stability. The morphology of the sprayed droplets under various conditions was systematically analyzed by optimizing parameters such as flow rate, outlet size, and applied voltage. Additionally, the electrospray process was enhanced by employing a moving substrate, which enabled the uniform deposition of droplets in an array format.

The uniformity and stability of the droplet array were validated using multiple analytical techniques, including fluorescence imaging, SEM, Raman spectroscopy, AFM, and KPFM. These analyses confirmed the consistency of droplet size and spatial distribution, highlighting the system's reliability for precision applications. Importantly, the use of liquid metal as an integrated electrode material significantly improved fabrication efficiency. Unlike conventional metal deposition methods that require multilayer lithography or bonding steps, the LM‐ESD system employs direct injection of liquid metal into prefabricated microchannels, simplifying the fabrication process while maintaining robust electrical functionality. This streamlined approach enhances scalability and reduces manufacturing costs.

Furthermore, the LM‐ESD system holds potential for diverse applications, including thin‐film coatings, single‐cell analysis, and high‐precision material deposition. By eliminating the need for external electrode assemblies while maintaining stable electrospray performance, the LM‐ESD system offers a scalable, versatile platform suitable for both advanced scientific research and industrial implementation. Looking ahead, future work will focus on mechanical developments such as multiplexed channel integration and automated substrate handling to enhance robustness and throughput, as well as biochemical applications including single‐cell deposition and biomolecular array preparation, thereby extending LM‐ESD system from device engineering to practical bioanalytical use.

## 4 Experimental Section

### Materials and Reagents

Silicon dioxide wafers (4‐inch) were fabricated using conventional lithography techniques. Polydimethylsiloxane (PDMS) was prepared by mixing the elastomer base and curing agent (Sylgard 184, Dow Corning) in the recommended ratio. Gallium‐indium eutectic alloy (Ga 75.5% / In 24.5%, ≥99.99% trace metals basis), sodium hydroxide solution (1 m, for HPCE), Rhodamine 6G (R6G, dry content ≈95%, powder), methanol (≥99.9%, suitable for immunofluorescence, HPLC), and acetic acid (≥99.7%) were all purchased from Sigma‐Aldrich. 3M Novec 7500 Engineered Fluid (Novec 7500 oil) and 3M Novec 1750 Engineered Fluid were provided by 3 m. FluoSurf S (5% w/w in Fluo‐Oil 135) was purchased from Emulseo (Bordeaux, France).

### LM‐ESD System Design and Fabrication

The chip was composed of three PDMS layers, two of which were spin‐coated and one cast‐molded. These layers were individually prepared and then bonded using oxygen plasma treatment (100 W for 1 min) to activate the surface and ensure strong adhesion. The embedded liquid metal was directly injected into prefabricated channels using a syringe. The PDMS mixture was prepared by combining the elastomer base and curing agent at a 10:1 ratio and cured on a hotplate (LK Lab, Korea) at 50 °C for 12 h.

The bottom layer was fabricated by pouring ≈1.6 g of the PDMS solution into a 60 mm Petri dish and spin‐coating at 500 rpm for 2 min. The middle layer, which contained the microfluidic channels, was fabricated on a 4‐inch silicon wafer. Approximately 3.2 g of the PDMS solution was poured onto the wafer and spin‐coated at 200 rpm for 2 min. The combined thickness of the bottom and middle layers was less than 200 µm.

The top layer was prepared by curing PDMS without spin‐coating to achieve a thickness of 5 mm, which was necessary for securely attaching tubing and introducing solutions into the microfluidic chip. A shallow groove was incorporated into the top layer design during molding, serving as a prefabricated cutting guide for shaping the pointed outlet region. The PDMS chip featured a groove that functioned as a cutting guide for the razor blade, enabling consistent and accurate sectioning while preventing accidental damage to the internal microchannels (Figure S10, Supporting Information).

To render the inherently hydrophilic PDMS surface hydrophobic, Novec 1750 fluid was injected into the microfluidic channels, and the chip was heated on a hotplate at 130 °C for 10 min. Following surface treatment, liquid metal (EGaIn, a gallium‐indium eutectic alloy) was injected into designated channels to form integrated electrodes for electrospraying. Prior to liquid metal injection, a sodium hydroxide solution was introduced into the channels to enhance wetting and facilitate flow. Following LM injection, the chip was briefly placed at –20 °C for 10 min to facilitate LM stabilization. Thereafter, the device was stored and operated under ambient conditions (23–25 °C).

### Microfluidic Droplet Generation

The droplets contained R6G, which was dissolved in the dispersed phase (methanol:acetic acid = 3:1, v/v), while Novec 7500 fluid mixed with 2% FluoSurf S served as the continuous oil phase. The R6G concentration was 20 µM, and the solution was prepared using an immobilizer consisting of a 3:1 mixture of methanol and acetic acid. Both the oil and R6G solutions were injected through a microfluidic pressure‐based flow controller (Fluigent, Le Kremlin‐Bicêtre, Île‐de‐France), with the pump operating in mbar units. The microfluidic chip was connected to the solution reservoirs via tubing, and the injection was driven by air pressure from the pump. To analyze the droplets, fluorescence imaging was performed using a fluorescence microscope (Nikon, Japan) equipped with a bright‐field and TRITC filter. The images were captured using a digital camera (EOS R6 Mark II, Canon, Japan) (Figure 2).

### Operation of LM‐ESD System

To operate the LM‐ESD system, an ITO glass substrate (2 Ω/sq) was used as the conductive base, which was essential for the electrospray process. A low‐noise, compact high‐voltage power supply (ES Series, +5 kV, Matsusada, Japan) was used to apply a positive bias voltage ranging from 0 to +5 kV to the ITO substrate to facilitate electrospraying. For array‐based spraying, both the microfluidic chip and ITO substrate needed to be securely fixed and capable of controlled movement. In this study, the ITO substrate was designed to move horizontally via an Arduino‐controlled motor installed beneath it. This motor operated with a DC 10 V power supply and was connected through a control cord. To analyze the electrospray pattern, a digital camera (A7S3, Sony, Japan) equipped with a 10× magnification lens (Nikon, Japan) was used (Figures 3 and 4).

### Fluorescence Microscope and SEM Analysis

Fluorescence microscopy was used to examine the fluorescence characteristics of R6G within droplets. Imaging was performed using a fluorescence stereomicroscope (SZX16, Olympus, Tokyo, Japan) equipped with an RFP filter (SZX2‐FRFP1, Olympus) for R6G detection. Fluorescence images were acquired at 3.2× magnification, and the spatial distribution and intensity of R6G fluorescence within the droplets were analyzed. A camera (EOS R6 Mark II, Canon, Japan) was used for image acquisition with the exposure time set to 10 s and ISO set to 640. These settings were optimized to minimize photobleaching while enhancing the signal clarity. Image processing and quantitative analysis were performed using ImageJ software. For SEM image analysis, the R6G located at the edge of the droplet was studied using field‐emission scanning electron microscopy (FE‐SEM; JSM‐7900F, JEOL, Tokyo, Japan). Images were obtained using SEM at 5 kV HV at various magnifications.

### KPFM Analysis

For atomic force microscopy (AFM) and Kelvin probe force microscopy (KPFM), droplet array sampling was performed on electrically conductive substrates, specifically ITO‐coated glass and gold‐coated glass. The droplet array was analyzed both electrically and topographically using the amplitude‐modulated KPFM mode of the AFM. Measurements were conducted in lift‐scan mode, based on the tapping mode, at 24 °C under ambient conditions. Pt‐coated conductive atomic force microscopy (AFM) probes (SCM‐PIT‐V2, Bruker) were used. In the initial scan, topographical imaging was performed in tapping mode with zero‐tip bias. During the subsequent lift scan, the AFM tip was raised 50 nm above the sample surface, and an alternating current (AC) bias voltage (V_AC_ = 500 mV) was applied at the cantilever's mechanical resonance frequency. The mechanical drive to the cantilever was disabled during the lift scan to facilitate the surface potential measurements. The scanning area was set to 40 µm × 40 µm, with a resolution of 512 samples/line. For optimal signal acquisition, the integral gain was adjusted to 10, the proportional gain to 30, and the amplitude set point to 1 nm. The obtained data were analyzed using Mountains SPIP software (version 9; Digital Surf, France) to obtain insights into the electrical properties of the R6G droplet arrays.

### Raman Analysis

All the Raman spectra were collected using a Renishaw inVia Raman microscope (inVia Reflex, Renishaw, Wotton‐under‐Edge, UK). A 785 nm wavelength laser was focused using a ×100 object lens (Leica DM2700 M, Germany, and Renishaw Centrus Detector, Wotton‐under‐Edge, UK). The spectra were measured using a 785 nm laser with a power of 11.50 mW after 1 s of exposure, with three spectra collected for local measurements and five for global mapping. Increased accumulation for global mapping was used to compensate for the broader scanning area, which could introduce more impurities and errors. All spectra were acquired in the range of 497–1622 cm^−1^. Cosmic rays were removed from each spectrum, and the polynomial baseline was subtracted using WiRE 5.1 software. Additionally, for the 3D plot, preprocessing was performed before the results were obtained. Based on the raw data, percentile‐based clipping (20th to 99th percentile) and standard deviation filtering (mean ± 2σ) were used to remove outliers. After removing the outliers, min–max normalization was applied to scale the Z values between 0 and 1 to ensure consistent data representation.

### Statistical Analysis

Curve fitting and statistical analysis were performed using GraphPad Prism 9. Spray angle–distance relationships were analyzed by linear regression, while Taylor cone size–distance relationships were analyzed using a sigmoidal (four‐parameter logistic) model to capture saturation behavior. For nozzle size comparison, spray angle data were fitted with linear regression, whereas Taylor cone size at the 30 µm outlet was analyzed with the sigmoidal model. In contrast, the Taylor cone size under the 20 µm outlet condition showed no meaningful dependence on distance and was therefore summarized as mean ± 95% CI without curve fitting.

## Conflict of Interest

The authors declare no conflict of interest.

## Author Contributions

C.K., S.R., and H.P. contributed equally to this work. C.K. performed the experiment, methodology, and writing of the original draft. S.R. carried out data curation, formal analysis, and writing of the original draft. H.P. worked on data curation, formal analysis, and writing of the original draft. S.L. conducted the experiment and worked on software. T.L. contributed to formal analysis and investigation. S.L. also contributed to investigation and data curation. H.K. performed validation and software. J.P. handled funding acquisition, supervision, and project administration. G.L. contributed to supervision and writing—review and editing. I.P. contributed to supervision, project administration, funding acquisition, and writing—review and editing. All authors read and approved the final paper for submission

## Supporting information



Supporting Information

Supplemental Movie 1

Supplemental Movie 2

Supplemental Movie 3

Supplemental Movie 4

Supplemental Movie 5

Supplemental Movie 6

## Data Availability

The data that support the findings of this study are available from the corresponding author upon reasonable request.
