# Supplementary material for: Needle‐free, Liquid Metal‐embedded Electrospray Deposition System for Controlled Microdroplet Printing
Source: Adv Sci (Weinh). 2025 Sep 26;13(3):e10905. doi: 10.1002/advs.202510905 (PMC12806309; doi:10.1002/advs.202510905)
Supplement: Supplementary file 1 — Supporting Information [file ADVS-13-e10905-s001.docx]

Supporting Information

Needle-free, Liquid Metal-embedded Electrospray Deposition System for Controlled Microdroplet Printing

Chaeyeong Kang^1^, Seokbeom Roh^2,3^, Hyunjun Park ^4^, Sugeun Lee^5^, Taeha Lee^2,3^, Sang Won Lee^5^, Hansung Kim^5^, Jinsung Park^1,4^*, Gyudo Lee^2,3,^*, Insu Park^5,^*

^1^ Department of MetaBioHealth, Sungkyunkwan University, Suwon 16419, Republic of Korea

^2^ Department of Biotechnology and Bioinformatics, Korea University, Sejong 30019, Republic of Korea

^3^ Interdisciplinary Graduate Program for Artificial Intelligence Smart Convergence Technology, Korea University, Sejong 30019, Republic of Korea

^4^ Department of Biomechatronics Engineering, Sungkyunkwan University, Suwon, 16419, Republic of Korea

^5^ Department of Biomedical Engineering, Yonsei University, Wonju 26493, Republic of Korea

**
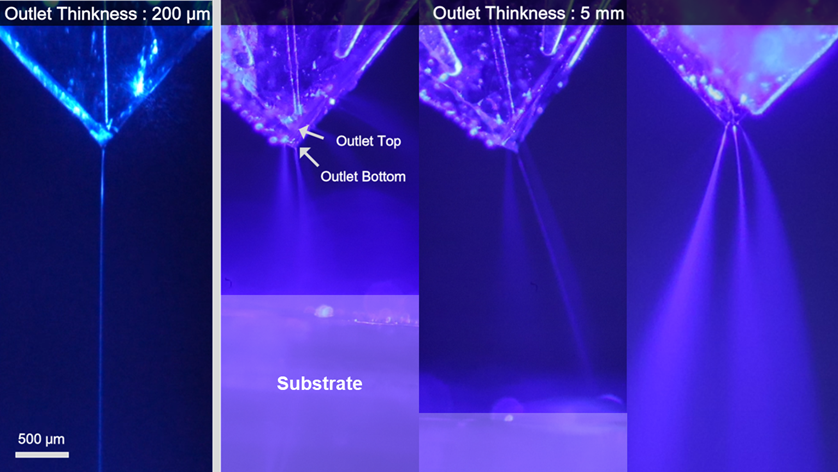
**

**Figure S1. Effect of outlet thickness on electrospray jetting behavior in the LM-ESD system.** When the outlet thickness is 200 µm (left), a single focused jet is observed. In contrast, increasing the outlet thickness to 5 mm (middle and right) results in multiple jet streams originating from both the top and bottom edges of the outlet, leading to a dispersed spray pattern on the substrate.


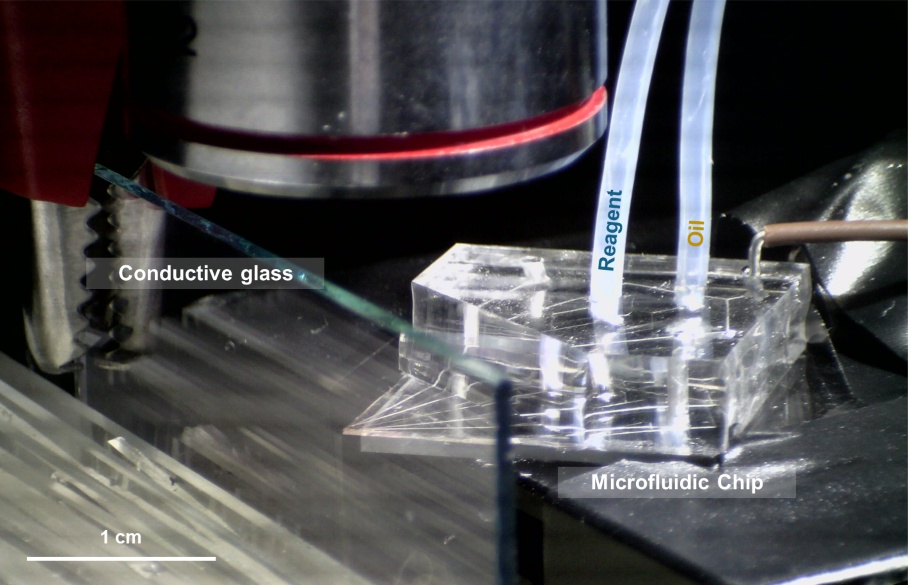


**Figure S2**. Actual stage image of a liquid metal-embedded electrospray deposition (LM-ESD) system. Reagents and oil were injected through an air pump and sprayed onto a conductive glass substrate (Scale bar: 1 cm).

**
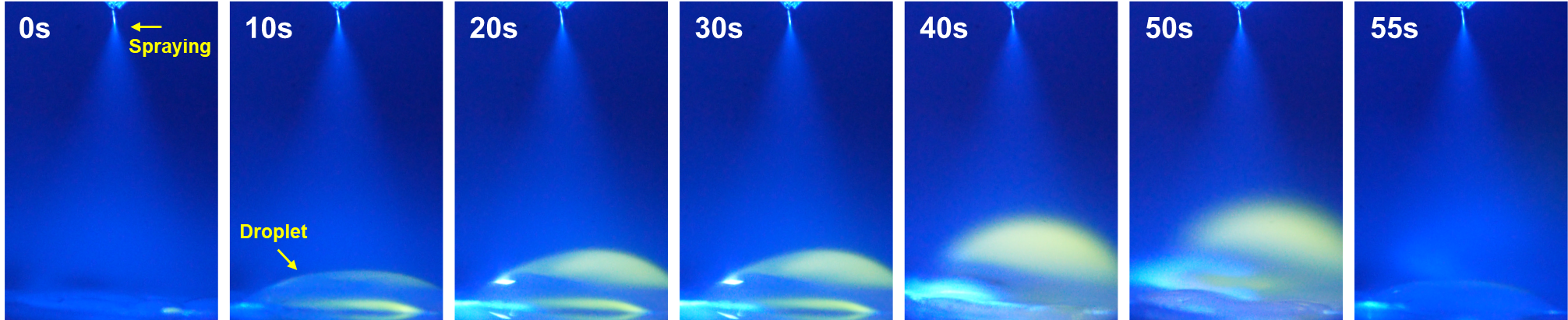
**

**Figure S3**. Continuous spraying in the LM-ESD system over time. During continuous spraying of the R6G solution, deposited droplets gradually coalesced into larger droplets. After 55 seconds, the coalesced droplets flowed off the substrate.

**Table S1. Type of Electrospray Systems**

|  | **Conductive needle** | **Surface electrode** | **External electrode** | **LM-ESD**  **(This work)** |
| --- | --- | --- | --- | --- |
|  | **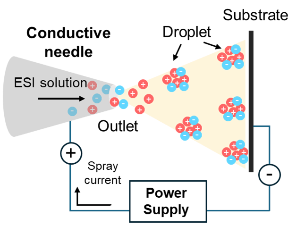** | **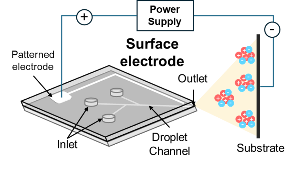** | **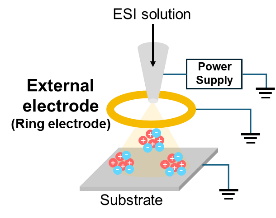** | **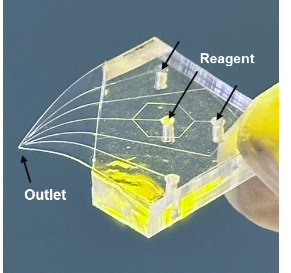** |
| **Electrode Integration** | Conductive needle manually inserted into the channel | Surface electrode patterned on substrate | External electrodes separate from the electrospray system | Integrating liquid metals into microfluidic chips |
| **Fabrication Complexity** | High: manual insertion and alignment needed | requires electrode fabrication and bonding | Moderate: requires precise alignment | Low: simple PDMS layer assembly and liquid metal injection |
| **Mechanical Robustness** | Low: susceptible to clogging and structural damage | Moderate: dependent on substrate thickness and bonding stability | Moderate: susceptible to delamination or misalignment | High: fully embedded design avoids fragile components and insertion |
| **Miniaturization potential** | Moderate | High | Low | High |
| **Reference** | **^[1,2]^** | **^[3,4]^** | **^[5,6]^** | **This work** |

**
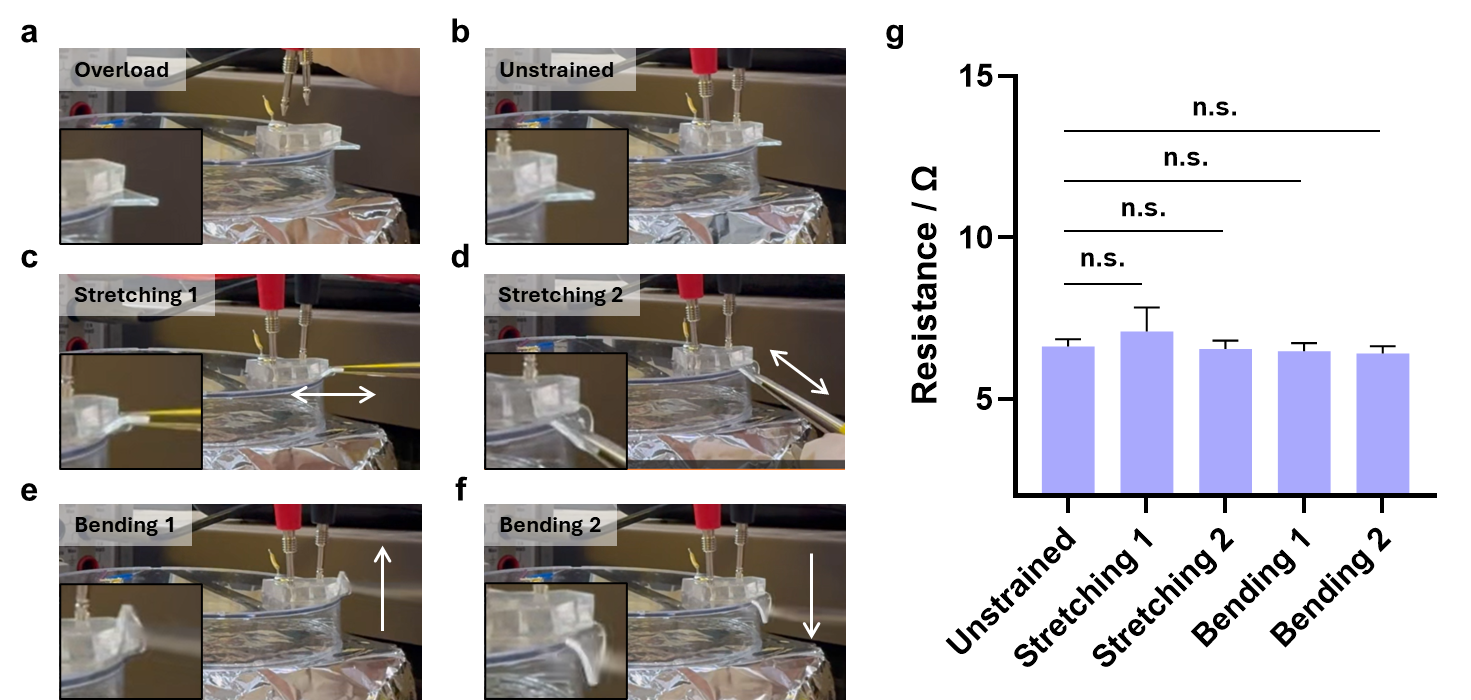
**

**Figure S4.** Resistance measurement of the liquid metal electrode in the LM-ESD system under mechanical deformation. a) before measurement, b) unstrained state without mechanical deformation, c), d) stretching conditions in various directions, e, f) vertical bending conditions. g) Resistance values of liquid metal EGaIn in the LM-ESD system showed no significant or statistically meaningful differences under various mechanical deformations, such as stretching and bending, compared to the normal state.

**
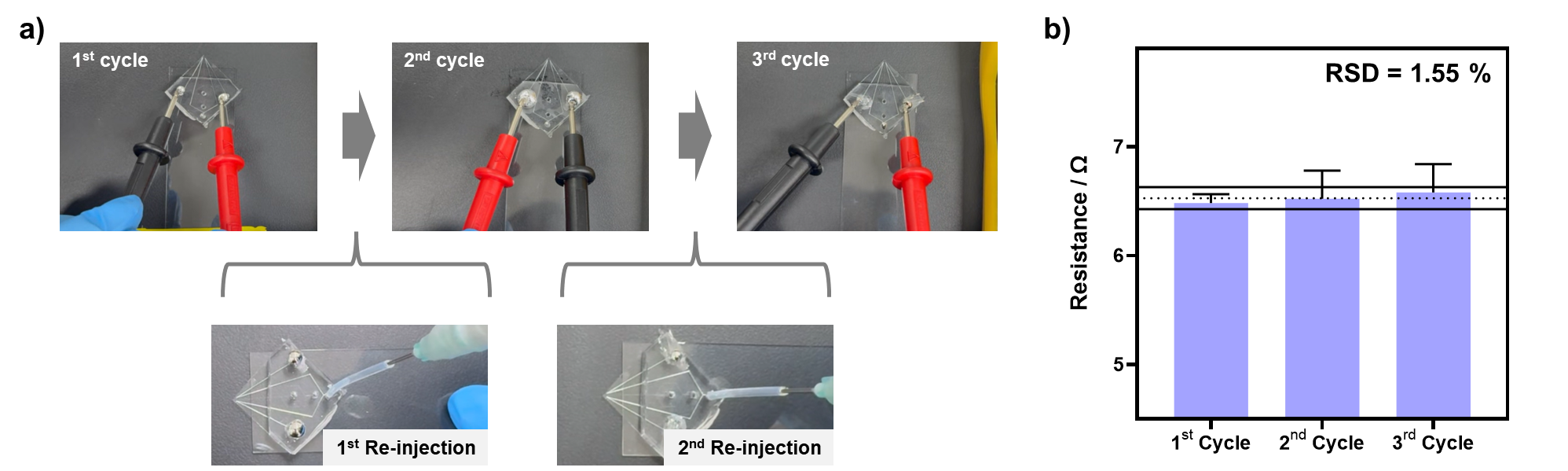
**

**Figure S5.** (a) Liquid metal reinjection cycles and electrode resistance measurements in the LM-ESD system. (b) Statistical summary of liquid metal resistance values ​​over multiple reinjection cycles (RSD=1.55%). This demonstrates the stable and reproducible performance of the built-in electrode. Error bars represent the standard deviation (n = 5).

**Table S2. Comparative Table: Prior Electrode Integration Methods vs. LM-ESD System**

| **System Type** | **PDMS chip with Electrolyte-filled Channel^[7]^** | **Glass Microchannel with Low-Melting Alloy Electrode^[8]^** | **PS–PDMS Hybrid with Embedded Electrode and Capillary Emitter^[9]^** | **Polymer Microchannel with Parylene Triangular Tip^[10]^** | **LM-ESD System** |
| --- | --- | --- | --- | --- | --- |
| **Electrode Configuration** | Electrolyte filled | Low-melting alloy (LMA) filled | Embedded palladium (rigid PS base) | External gold wire at channel reservoir | Liquid metal embedded (EGaIn) |
| **Emitter Structure** | PDMS-embedded sharp tip with external voltage | Monolithic glass with alloy electrodes and fused silica capillary | Integrated fused silica capillary pulled to ~30 µm tip | Lithographically patterned parylene triangular thin-film tip | Needle-free, fully molded PDMS microstructure |
| **Spray Mode** | ESI cone-jet | ESI cone-jet | ESI cone-jet | ESI cone-jet | ESI cone-jet & on-demand transition between droplet formation |
| **Application Direction** | Direct MS coupling | HPLC-MS interface | Direct MS coupling | Multiplexed ESI-MS | Precision droplet patterning for bioassay, array fabrication |
| **External Components** | External Au electrode | Requires external alloy injection and alignment | No external wiring; electrode embedded in PS | External Au electrode | Operates solely with internal electrodes (no external HV wiring) |


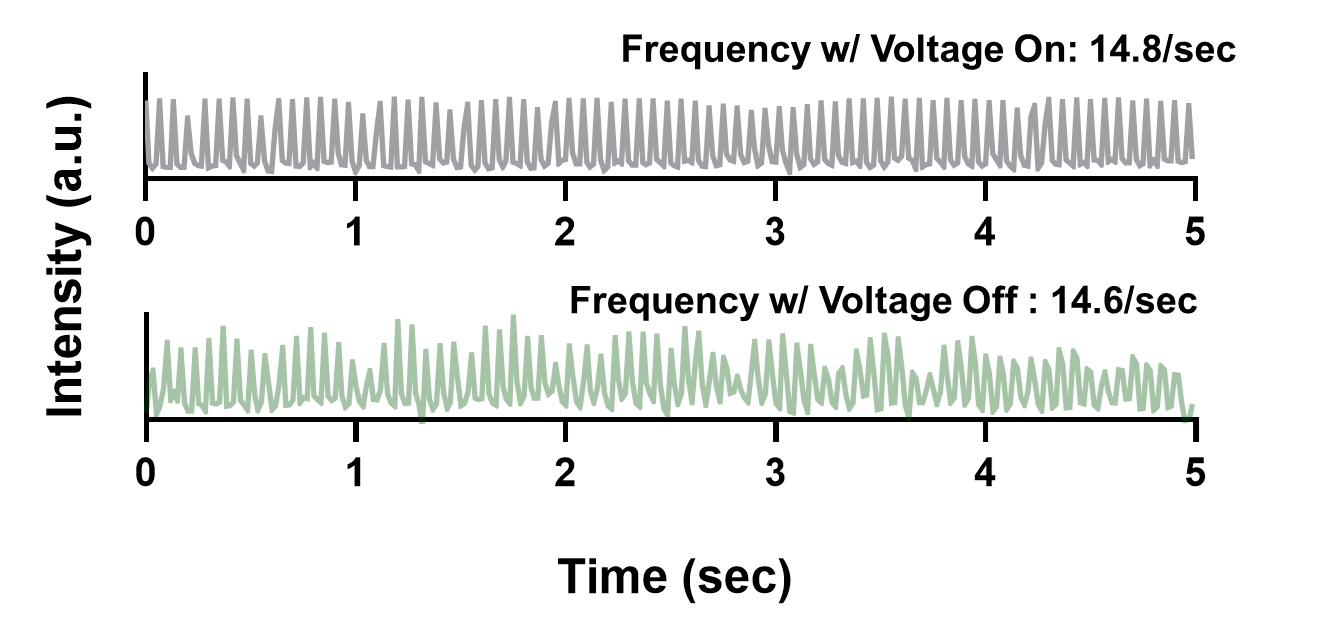


**Figure S6.** Comparison of droplet generation frequencies with and without applied voltage. Time-trace intensity profiles demonstrate consistent periodic droplet formation at frequencies of 14.8 s⁻¹ (voltage on, top) and 14.6 s⁻¹ (voltage off, bottom). The negligible difference in droplet frequency confirms that the embedded LM electrodes do not perturb droplet generation dynamics, thereby validating stable electrospray operation under applied high-voltage conditions.


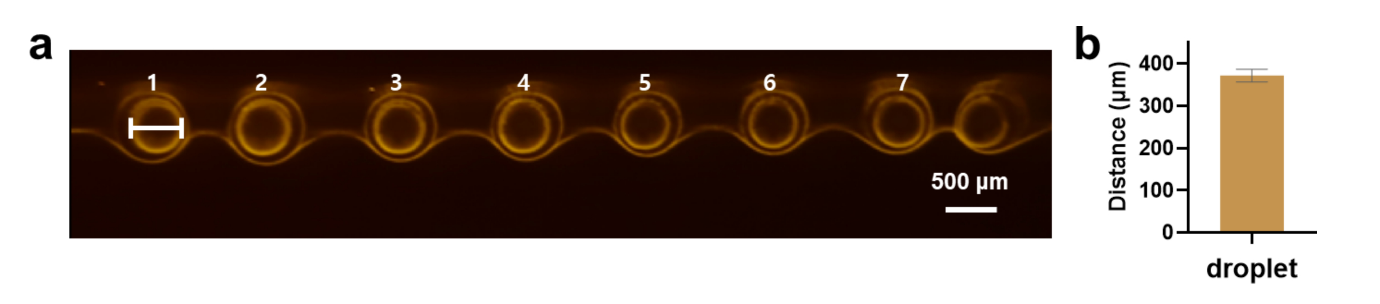


**Figure S7**. Droplet size analysis through fluorescence images. (a) Fluorescence image showing a linear array of seven individual droplets generated by the LM-ESD system on an ITO-coated glass (b) Quantitative measurement of the droplet diameter corresponding to droplets 1 through 7 in (a), based on the fluorescence edge profiles. The results demonstrate high consistency in droplet generation across the array.

**S8. Topography and surface potential of LM-ESD-deposited droplet**


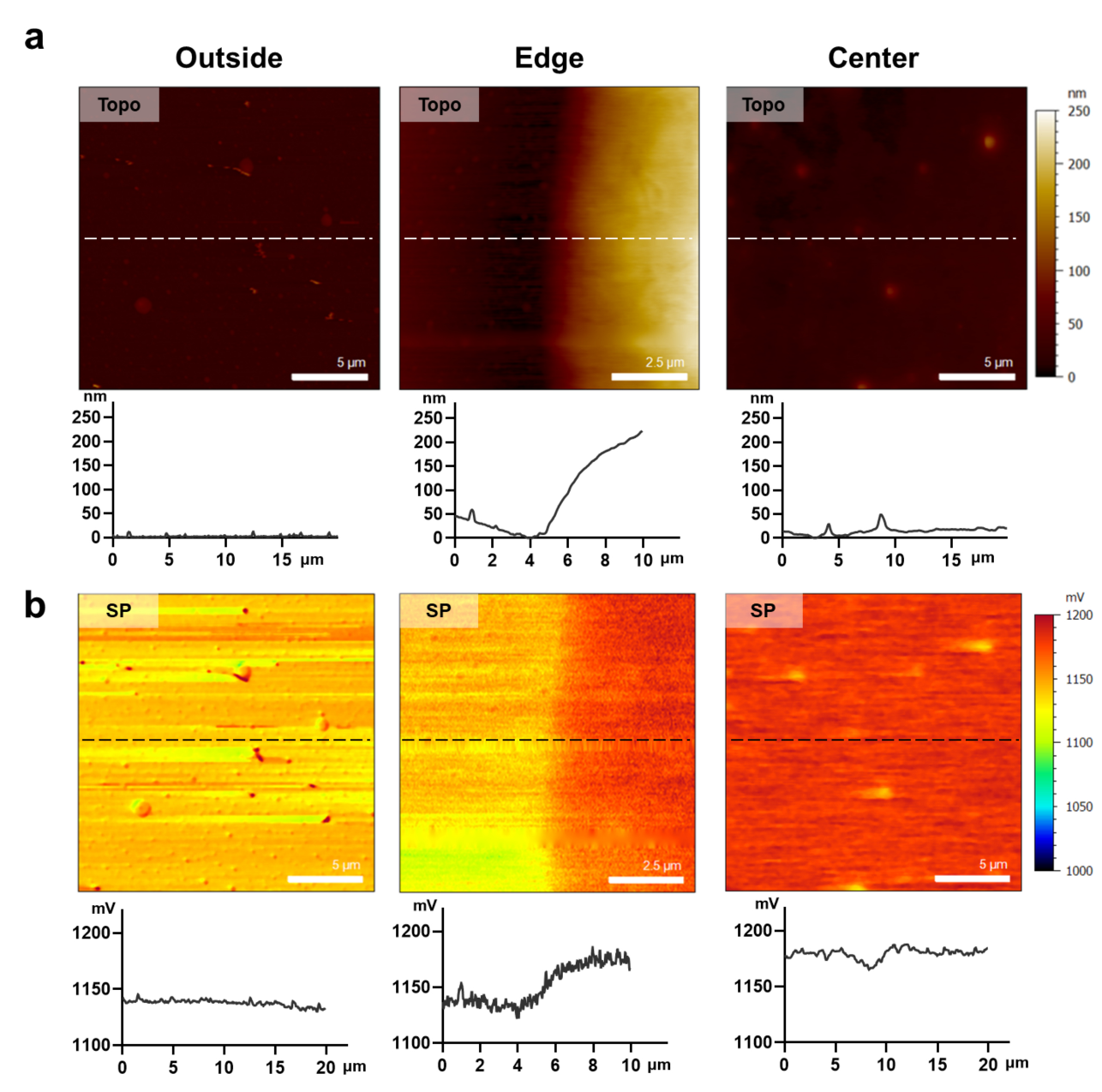


**Figure S8**. Topography and surface potential mapping of R6G droplet on a gold substrate using KPFM. (a) Topographic images of three representative regions—outside, edge, and center—within a single droplet showing a clear height increase at the edge, consistent with coffee-ring-type accumulation during solvent evaporation. The corresponding cross-sectional profile (dashed white lines) reveal a ridge-like structure at the droplet edge, while the center and outside regions remain relatively flat. (b) Surface potential maps of the same regions exhibit an overall elevation in potential within the droplet area compared to the surrounding substrate. However, the potential values at the edge and center are similar, indicating no significant electrostatic localization at the edge. The cross-sectional profile (dashed black lines) highlights the uniformity in potential across the droplet, suggesting redistribution or partial neutralization of charge after deposition.

The combined topographic and surface potential mapping of the LM-ESD-deposited R6G droplets revealed nuanced decoupling between material accumulation and surface charge distribution. While AFM measurements clearly demonstrated the peripheral build-up of material at the droplet edge, forming a characteristic ridge structure attributed to the coffee-ring effect, KPFM analysis showed no significant potential difference between the edge and center regions. Both regions exhibited an elevated surface potential relative to the surrounding substrate but lacked strong edge-specific charge localization.

This finding suggests that, although the LM-ESD system effectively deposited ionized droplets, the resulting charge may be uniformly redistributed across the droplet footprint or dissipated upon contact with the conductive gold substrate. The absence of electrostatic asymmetry at the edge, despite concentrated molecular accumulation, highlights the role of post-deposition charge relaxation mechanisms and substrate interactions in determining the final surface potential landscape. More broadly, this result emphasizes the importance of multimodal characterization of electrospray-based deposition systems. Morphological or chemical gradients were not fully captured in the charge behavior of the ionized droplets, especially when the charge retention was influenced by surface conductivity or drying dynamics.

**S9. Raman Analysis of Droplet Arrays**


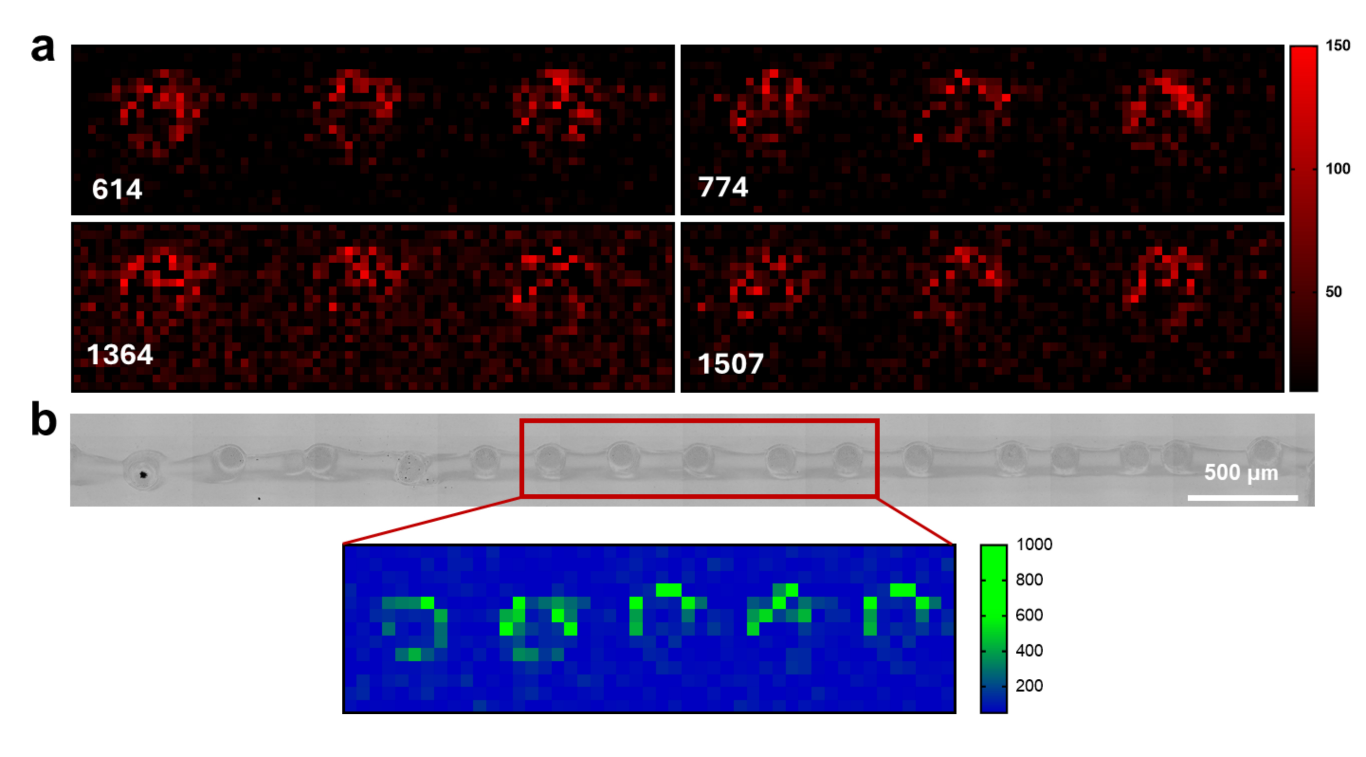


**Figure S9**. Raman spectroscopic mapping images of R6G droplets (a) Mapping results based on distinct Raman peaks (614 cm^-1^, 774 cm^-1^, 1364 cm^-1^, and 1,507 cm^-1^) of R6G on an ITO conductive substrate. (b) Optical and corresponding Raman mapping images of droplets deposited on a gold substrate. The coffee-ring effect is consistently observed on both types of substrates.

Using an LM-ESD system, we analyzed the array of droplets by spraying R6G onto an indium tin oxide (ITO) substrate, a conductive electrode. Figure S9a shows four representative images of the sprayed droplets, and each shows a mapping image according to the intensity, based on the characteristic Raman peaks of R6G (614, 774, 1364, and 1,507 cm^-1^). We confirmed that the higher intensity at the edge of the droplet than at the center was due to the coffee-ring effect that appeared in the droplets. Subsequently, 17 consecutive spray arrays were confirmed on the gold substrate using an optical image (Figure S9b), and the uniformity of the spray deposition was experimentally verified by confirming the Raman mapping image in a specific area.


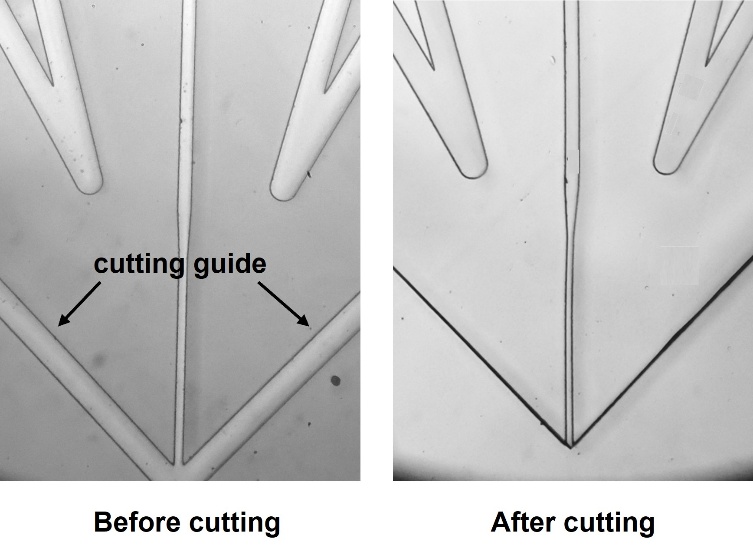


**Figure S10.** Fabrication of the pointed outlet region using a predefined cutting guide. (Left) PDMS outlet region before cutting, showing the integrated shallow groove that serves as the cutting guide. (Right) Outlet region after cutting along the guide using a razor blade, resulting in a pointed geometry. The PDMS chip featured a groove that functioned as a cutting guide for the razor blade, enabling consistent and accurate sectioning without damaging the internal microchannels.

**Movie S1. Effect of Outlet Thickness on Jetting Behavior in the LM-ESD System**

This video demonstrates how the outlet thickness of the fabricated LM-ESD system affects droplet jetting behavior. When the outlet thickness was increased to 5 mm, the system exhibited a multijet ejection mode rather than a cone jet. This highlights the critical role of the outlet thickness in determining the electric field distribution and jetting stability during the electrospray operation.

**Movie S2. Solving Clogging Problems in an LM-ESD System Through Flexibility**

This video demonstrates that when clogging occurs, simply pulling the flexible substrate can effectively remove the clogging. This demonstrates a simple yet effective solution that maintains stable operation during the electrospray process and easily resolves clogging issues.

**Movie S3. Visualization of droplet size variations under different pressure conditions in the oil and R6G dispersed phases.**

Notably, as the pressure of the R6G solution increased, the size of the ejected droplets correspondingly increased, indicating the pressure-dependent modulation of droplet formation dynamics.

**Movie S4. Effect of nozzle-substrate distance on the size of the Taylor cone.**

A clear increase in cone size was observed as the substrate approached the nozzle of the LM-ESD system, demonstrating that shorter distances promoted the formation of larger Taylor cones because of the enhanced electric field concentration.

**Movie S5. Observation of electrospray behavior at 2 and 4 kV applied voltages.**

At both voltages, only the oil phase was successfully sprayed, whereas the R6G solution phase failed to emit droplets, and instead retracted into the microchannels. These results support the definition of a non-spraying regime below 4.5 kV, as shown in Figure 4e, where water-based droplets were suppressed despite the possibility of oil-only spraying.

**Movie S6. Long-Term Stability of the LM-ESD System**

This video demonstrates the exceptional long-term stability of the system. The electrospray operation remains consistently stable, continuing without interruption until the reagent is fully consumed. This highlights the system's robust performance and its ability to maintain stable operation for extended periods.

**Reference**

1 Zhao, Y. *et al.* 1-Octanol-assisted ultra-small volume droplet microfluidics with nanoelectrospray ionization mass spectrometry. *Analytica chimica acta* **1321**, 342998 (2024).

2 Cech, N. B. & Enke, C. G. Practical implications of some recent studies in electrospray ionization fundamentals. *Mass spectrometry reviews* **20**, 362-387 (2001).

3 Peretzki, A. J. *et al.* How electrospray potentials can disrupt droplet microfluidics and how to prevent this. *Lab on a Chip* **20**, 4456-4465 (2020).

4 Feng, D. *et al.* High-throughput single cell metabolomics and cellular heterogeneity exploration by inertial microfluidics coupled with pulsed electric field-induced electrospray ionization-high resolution mass spectrometry. *Analytica Chimica Acta* **1221**, 340116 (2022).

5 Jaworek, A. Electrospray droplet sources for thin film deposition. *Journal of materials science* **42**, 266-297 (2007).

6 Chua, Z. Q. *et al.* Moderate Signal Enhancement in Electrospray Ionization Mass Spectrometry by Focusing Electrospray Plume with a Dielectric Layer around the Mass Spectrometer’s Orifice. *Molecules* **29**, 316 (2024).

7 Kelly, R. T., Tang, K., Irimia, D., Toner, M. & Smith, R. D. Elastomeric Microchip Electrospray Emitter for Stable Cone-Jet Mode Operation in the Nanoflow Regime. *Analytical Chemistry* **80**, 3824-3831, doi:10.1021/ac8000786 (2008).

8 Zhu, Y., Pan, J.-Z., Su, Y., He, Q.-H. & Fang, Q. Fabrication of low-melting-point alloy microelectrode and monolithic spray tip for integration of glass chip with electrospray ionization mass spectrometry. *Talanta* **81**, 1069-1075, doi:<https://doi.org/10.1016/j.talanta.2010.01.064> (2010).

9 Forzano, A. V., Becirovic, V., Martin, R. S. & Edwards, J. L. Integrated Electrodes and Electrospray Emitter for Polymer Microfluidic Nanospray-MS Interface. *Anal Methods* **8**, 5152-5157, doi:10.1039/c6ay00197a (2016).

10 Kameoka, J. *et al.* An Electrospray Ionization Source for Integration with Microfluidics. *Analytical Chemistry* **74**, 5897-5901, doi:10.1021/ac020396s (2002).
